# Current Status of Diagnostic Process in Asymptomatic Abdominal Aortic Aneurysm in Japan

**DOI:** 10.3400/avd.oa.25-00025

**Published:** 2025-03-28

**Authors:** Yoshimasa Seike, Nobuyoshi Azuma, Takao Ohki, Noriyasu Morikage, Akio Kodama, Makoto Sumi, Koji Maeda, Hitoshi Matsuda

**Affiliations:** 1 Department of Cardiovascular Surgery, National Cerebral and Cardiovascular Center, Suita, Osaka, Japan; 2 Department of Vascular Surgery, Asahikawa Medical University, Asahikawa, Hokkaido, Japan; 3 Division of Vascular Surgery, Department of Surgery, The Jikei University School of Medicine, Tokyo, Japan; 4 Division of Vascular Surgery, Department of Surgery and Clinical Science, Yamaguchi University Graduate School of Medicine, Ube, Yamaguchi, Japan; 5 Department of Vascular Surgery, Aichi Medical University, Nagakute, Aichi, Japan; 6 Department of Vascular Surgery, International University of Health and Welfare Hospital, Nasushiobara, Tochigi, Japan; 7 Department of Vascular Surgery, International University of Health and Welfare Narita Hospital, Narita, Chiba, Japan

**Keywords:** abdominal aortic aneurysm, screening, computed tomography, abdominal ultrasonography, multicenter study

## Abstract

**Objectives:** This study aimed to investigate the actual detection process and diagnostic methods for asymptomatic abdominal aortic aneurysm (AAA) in a multicenter setting, and to plan an effective screening strategy for asymptomatic AAA.

**Methods:** The subjects of this multicenter study were collected in a retrospective manner at 7 facilities. A total of 1894 patients with AAA, including iliac artery aneurysms, who were considered asymptomatic with a confirmed initial diagnosis from January 2018 to December 2022, were collected and reviewed.

**Results:** A total of 1666 patients who were diagnosed with asymptomatic AAA were included [83.9% males, median age of 75 (69–81) years]. Asymptomatic AAAs were frequently diagnosed during examinations for other diseases in 1339 patients (80.4%), whereas health screenings accounted for only 313 (18.8%). Computed tomography (CT) was the most commonly used diagnostic method (n = 1352, 81.2%) compared to abdominal ultrasonography (n = 252, 15.2%).

**Conclusions:** Asymptomatic AAAs are detected incidentally during examinations for other diseases, and there is an urgent need to promote health screening. Most AAAs are diagnosed by CT; nevertheless, we consider that abdominal ultrasonography would be the most appropriate modality for AAA screening because of its reasonable accuracy, noninvasiveness, and low cost.

## Introduction

In Japan, with a population of 124.5 million, national vital statistics from the Ministry of Health, Labor, and Welfare (MHLW) reported about 3000 deaths annually after abdominal aortic aneurysm (AAA) rupture. The annual report from the Japanese Society for Vascular Surgery (JSVS) reported 1719 surgeries for AAA rupture in 2016 and 286 operative deaths. As operative deaths are included in the deaths after AAA rupture in vital statistics from MHLW, an approximate 70% mortality rate (3000 deaths/ [3000 + 1719 − 286] AAA ruptures = 68%) after the onset of AAA rupture is expected. Also, the chance of arriving at the operating theater after an AAA rupture is calculated to be around 40% (1719 surgeries/ [3000 + 1719 − 286] AAA ruptures = 39%).

JSVS annual report also stated improved hospital mortality of 0.6% after scheduled surgery and 15.7% after surgeries for AAA rupture.^1)^ Although the life-saving rate after AAA rupture is improving with advancements in imaging technology and a well-organized emergency medical system, a significant gap in surgical results remains between scheduled and emergency surgeries for AAA.

After the launch of commercially available stent grafts for endovascular aneurysm repair (EVAR) in 2005, EVAR has already been accepted as a usual option to treat AAA, and the operative indication for senescent patients was extended. To improve the comprehensive surgical outcome of AAA, it is essential to diagnose AAA before rupture and indicate suitable surgical procedures, replacement through laparotomy or EVAR, for each patient according to the aneurysm diameter recommended in the Japanese guideline.^2)^

AAA screening has been implemented in the United States and Europe; nevertheless, it is not as common in Japan, despite the prevalence of cancer screening and lifestyle health check-ups.^3–5)^ Baba described that in 1500 patients who underwent EVAR, the initial diagnosis was made after the diagnostic process for other diseases in 68%, after health check-ups in 22%, and as a differential diagnosis for abdominal pain in 10%.^6)^ This data would likely be agreed upon by the majority of vascular surgeons in Japan, who recognize in daily clinical practice that the majority of patients with AAA are usually referred after a diagnosis made by chance.

This study investigated the actual diagnostic process methods of asymptomatic AAA in a multicenter setting and aimed to plan an effective screening strategy for asymptomatic AAA in Japan.

## Materials and Methods

### Ethics statement and study design

This is a multicenter, retrospective, financially unsupported physician-initiated observational cohort study conducted by 6 academic tertiary referral hospitals in Japan, and approved by the Institutional Review Board (R22061-2) at the National Cerebral and Cardiovascular Center, and local committee at each facility. Each patient’s oral and written informed consents were waived because of its retrospective design. Clinical data were collected in a retrospective manner at each facility.

### Study population

**Figure 1:**
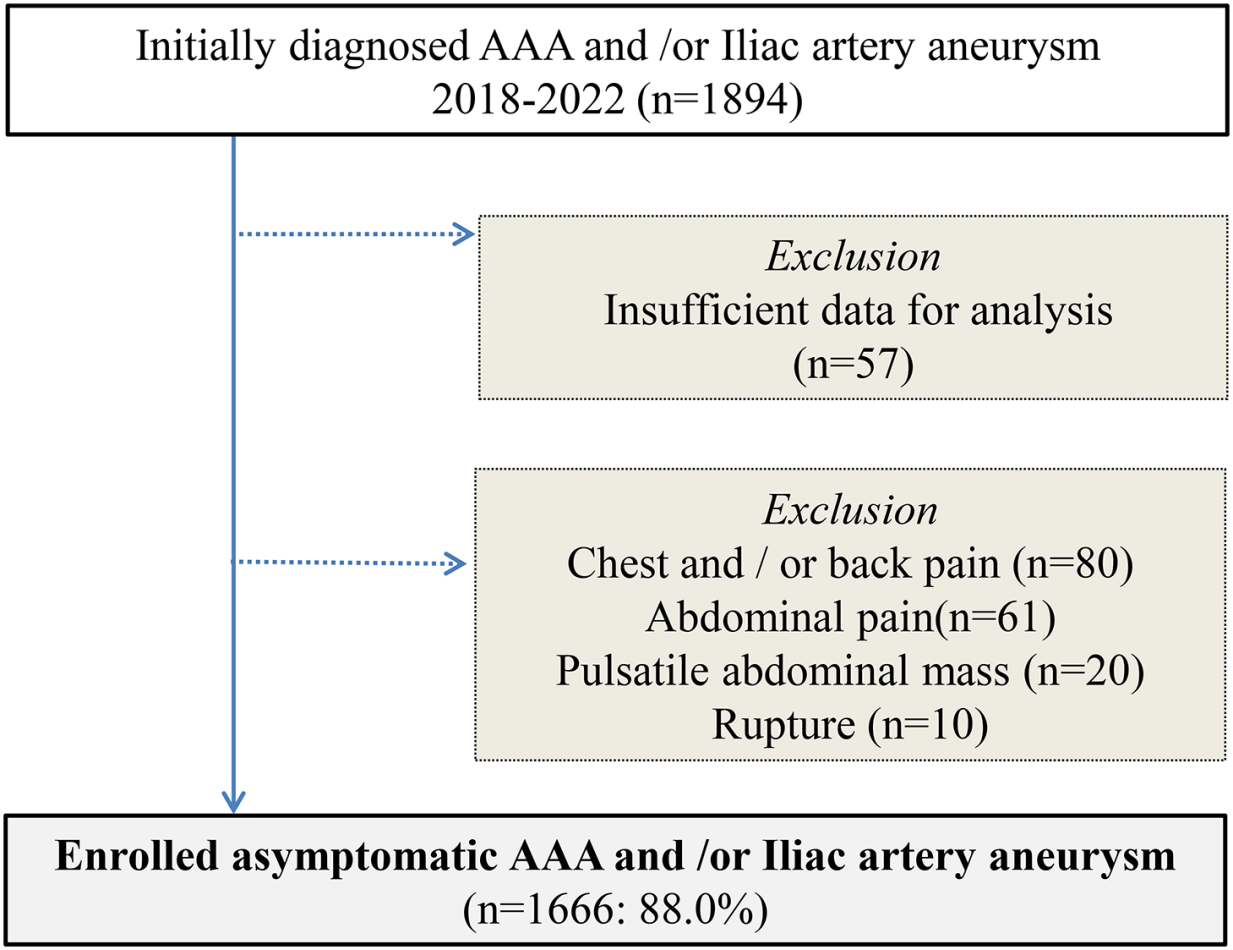
Fig. 1 Flowchart of study population and method. Based on the listed exclusion criteria, 1666 patients were enrolled in this study. AAA: abdominal aortic aneurysm

Data of 1894 patients in total with AAA and/or iliac artery aneurysm (IAA) who were considered asymptomatic with a confirmed initial diagnosis from January 2018 to December 2022 were collected from outpatient or hospital charts retrospectively and reviewed. To refine the target cohort and to properly evaluate the outcomes, 80 patients were excluded for chest and/or back pain, 61 for abdominal pain, 20 for awareness of pulsatile abdominal mass, 10 for aortic rupture, and 57 for insufficient data for analysis. As a result, 1666 patients with asymptomatic AAA and/or IAA (hereafter referred to as asymptomatic AAA) were enrolled (**Fig. 1**).

### Reporting items

Observations include the following:

(1) Data on initial diagnosis of asymptomatic AAA: date of diagnosis, age, sex, height, weight, presence/absence of emergency transport, medical history, family history, diagnostic method [abdominal ultrasonography (AUS), echocardiography, computed tomography (CT), magnetic resonance imaging (MRI), etc.], and the aneurysm’s maximum diameter measured by the diagnostic method.

(2) Follow-up data: (i) in patients with treatment for AAA: date of treatment, age at treatment, treatment procedure (graft replacement, EVAR), diagnostic method for measuring aneurysm diameter at the time of treatment (AUS, echocardiography, CT, MRI, etc.), and date of last known alive or date of death; (ii) in patients without treatment for AAA: date of last observation and date of last confirmed survival or date of death.

### Primary and secondary outcomes

The primary outcome was how to diagnose asymptomatic AAA at the time of the initial examination, including the purpose and method of diagnosis. The secondary outcomes were patient characteristics at initial diagnosis, the presence or absence of an indication for AAA repair at initial diagnosis, and freedom from all-cause mortality.

### Statistical analysis

Statistical analyses were conducted using Stata software (Stata Corp LLC, College Station, TX, USA). After assessing normality using the Shapiro–Wilk test, continuous variables were analyzed. All variables, including patient age, height, body weight, and aneurysm size, were expressed as the median and interquartile range. Categorical data were compared using the Fisher exact test. Continuous variables were compared using the nonparametric Mann–Whitney *U*-test. Survival rates were estimated using the Kaplan–Meier method, and differences between groups were determined by log-rank analysis. Values of p < 0.05 were considered statistically significant.

## Results

### Patients’ characteristics at initial diagnosis

The characteristics of 1666 patients are summarized in **Table 1**. The patients comprised 1398 men (83.9%) with a median age of 75 (69–81) years. The diameter of aneurysm at initial diagnosis was 44 (36–52) mm on average; less than 35 mm in 308 (18.5%) patients, 35 to less than 40 mm in 279 (16.7%), 40 to less than 45 mm in 270 (16.2%), 45 to less than 50 mm in 243 (14.6%), 50 to less than 55 mm in 220 (13.3%), and 55 mm or greater in 346 (20.7%). Smoking habits, including past smoking, were found in 1151 (69.1%) patients, hypertension in 1284 (77.1%), coronary artery disease in 386 (23.2%), chronic kidney disease (CKD) in 505 (30.3%), and family history of AAA in 76 (4.6%) (**Table 1**).

**Table:** Table 1 Patient characteristics and clinical features

Variables	Entire cohort (n = 1666)	Treated (n = 1188)	Untreated (n = 478)	p value
Male	1398 (83.9%)	1009 (60.6%)	389 (23.3%)	0.077
Age at diagnosis (years)	75 (69–81)	75 (69–81)	75 (69–81)	0.305
Height (cm)	164 (159–170)	164 (159–169)	165 (158–170)	0.146
Body weight (kg)	61 (53–70)	61 (54–70)	62 (53–70)	0.854
Aneurysm diameter at initial diagnosis (mm)	44 (36–52)	47 (40–55)	36 (32–42)	<0.001
Aneurysm <35	308 (18.5%)	129 (7.7%)	179 (10.8%)	–
35< Aneurysm <40	279 (16.7%)	143 (8.6%)	136 (8.1%)	–
40< Aneurysm <45	270 (16.2%)	193 (11.6%)	77 (4.6%)	–
45< Aneurysm <50	243 (14.6%)	196 (11.8%)	47 (2.8%)	–
50< Aneurysm <55	220 (13.3%)	203 (12.2%)	17 (1.1%)	–
Aneurysm <55	346 (20.7%)	324 (19.4%)	22 (1.3%)	–
Iliac artery aneurysm	138 (8.3%)	132 (7.9%)	6 (0.4%)	<0.001
Smoking	1151 (69.1%)	822 (49.3%)	329 (19.8%)	0.907
Hypertension	1284 (77.1%)	955 (57.3%)	329 (19.8%)	<0.001
Hyperlipidemia	768 (46.1%)	567 (34.0%)	201 (12.1%)	0.039
Diabetes mellitus	312 (18.7%)	230 (13.8%)	82 (4.9%)	0.331
Cerebrovascular disease	366 (22.0%)	275 (16.5%)	91 (5.5%)	0.067
Coronary artery disease	386 (23.2%)	307 (18.4%)	79 (4.8%)	<0.001
Chronic heart failure	96 (5.8%)	67 (4.1%)	29 (1.7%)	0.728
Chronic obstructive pulmonary disease	294 (17.8%)	201 (12.1%)	93 (5.7%)	0.227
Chronic kidney disease	505 (30.3%)	359 (21.5%)	146 (8.8%)	0.906
Arteriosclerosis obliterans	201 (12.1%)	157 (9.4%)	44 (2.7%)	0.025
Family history of AAA	76 (4.6%)	56 (3.4%)	20 (1.2%)	0.698

AAA: abdominal aortic aneurysm

In terms of differences between the patients with treatment for AAA (treated group: n = 1188, 71.3%) and the patients without treatment for AAA (untreated group: n = 478, 28.7%) during the study period, the aneurysm diameter at initial diagnosis was significantly larger in the treated group [47 (40–55) mm] than the untreated group [36 (32–42) mm] (p < 0.001) and IAA was more frequent in the treated group (7.9%) than the untreated group (0.4%) (p < 0.001). In terms of comorbidity, hypertension (57.3% vs. 19.8%, p < 0.001), hyperlipidemia (34.0% vs. 12.1%, p = 0.039), coronary artery disease (18.4% vs. 4.8%, p < 0.001), and arteriosclerosis obliterans (9.4% vs. 2.7%, p = 0.025) were more frequent in the treated group than the untreated group. There were no differences in the other variables between the 2 groups (**Table 1**).

### The diagnostic process

In 79.5% of patients, AAA was found by chance during examinations for other diseases (**Table 2**). As for other diagnostic purposes, health screenings accounted for 18.8%, including public health screening (7.3%), comprehensive medical examination (6.0%), and private health screening (5.5%) Among diagnostic methods, CT was most common, used in more than 80%, and limited utilization of AUS, at 15.2%, was observed.

**Table:** Table 2 The diagnostic process

Variables	No (%)
Purpose for diagnosis	
During examination of other diseases	1339 (80.4)
Details of “during examination of other diseases”	(% of other diseases)
Malignancy*	260 (19.4)
Gastrointestinal disease*	134 (10.0)
Ischemic heart disease	116 (8.6)
Orthopedic disease	107 (8.0)
Urological disease*	105 (7.9)
Biliary, hepatic, and pancreatic diseases*	94 (7.0)
Cerebrovascular disease	93 (6.9)
Respiratory disease	88 (6.6)
Thoracic aortic aneurysm/dissection	70 (5.2)
Peripheral vascular disease	52 (3.9)
Chronic kidney disease*	51 (3.8)
Valvular disease/arrhythmia	50 (3.8)
Collagen, endocrine, and metabolic diseases	35 (2.6)
Common cold symptoms	22 (1.6)
Dermatological disease	11 (0.8)
Hypertension	9 (0.7)
Venous and lymphatic disease	9 (0.7)
Ophthalmology, otolaryngology, and dentistry	6 (0.5)
COVID-19	5 (0.4)
Hematological disease	4 (0.3)
Gynecological disease*	2 (0.2)
Psychiatric disorders	1 (0.1)
Health screening	313 (18.8)
Public health screening	122
Comprehensive medical examination	100
Private health screening in hospital	91
Others	14 (0.8)
Method for diagnosis	
Computed tomography	1352 (81.2)
Abdominal ultrasonography	252 (15.2)
Magnetic resonance imaging	38 (2.3)
Echocardiography	19 (1.1)
Aortography	2 (0.1)
Others	2 (0.1)

*Abdominal organs. COVID-19: Corona virus disease-2019

**Figure 2:**
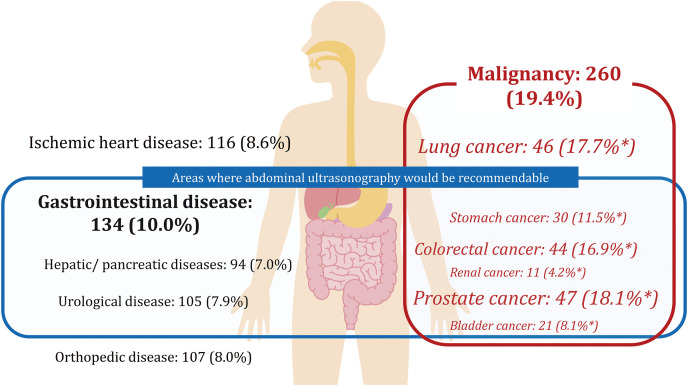
Fig. 2 Primary disease for examination which diagnosed abdominal aortic aneurysm. Malignancy was the most common (19.4%) disease. Among them, prostate cancer (18.1%), lung cancer (17.7%), colorectal cancer (16.9%), and stomach cancer (11.5%) occupied more than 60%. The asterisk (*) indicates occupancy within malignant disease.

Primary diseases for examination, which also diagnosed AAA by chance, are listed in **Table 2**. Malignancy was the most common (19.4%) disease, and among these 260 patients with malignancy, prostate cancer (18.1%), lung cancer (17.7%), colorectal cancer (16.9%), and stomach cancer (11.5%) accounted for more than 60%; This distribution is similar to the order of number of patients with malignancy in Japan: prostate, colorectal, lung, and stomach in males, and breast, colorectal, lung, and stomach cancers in females (**Table 3**). Among primary disease for examination, around 35% of diseases were located in the abdominal organs: abdominal non-malignancies (gastrointestinal disease in 10.0%, hepatic/pancreatic disease in 7.0%, and urological disease in 7.9%) and abdominal malignancies, which accounted for more than half of all malignancies (**Fig. 2**).

**Table:** Table 3 The details of malignancy (n = 260)

Variables	No (%)
Prostate cancer*	47 (18.1)
Lung cancer	46 (17.7)
Colorectal cancer*	44 (16.9)
Stomach cancer*	30 (11.5)
Bladder cancer*	21 (8.1)
Renal cancer*	11 (4.2)
Head and neck cancer	10 (3.8)
Hepatic cancer*	9 (3.5)
Breast cancer	9 (3.5)
Hematologic cancer	8 (3.1)
Skin cancer	5 (1.9)
Pancreatic cancer*	4 (1.5)
Uterine cancer*	4 (1.5)
Esophageal cancer	4 (1.5)
Ureteral cancer*	3 (1.2)
Gallbladder cancer*	1 (0.4)
Vulvar cancer	1 (0.4)
Ovarian cancer*	1 (0.4)
Cancer of unknown primary	2 (0.8)

*Abdominal organs

### Follow-up data

The mean follow-up time (months) was significantly longer in the treated group [36 (19–54)] than in the untreated group [19 (8–36)] (p < 0.001). During the study period in the treated group (n = 1188), 245 (20.6%) patients were treated by graft replacement and 943 (79.4%) patients by EVAR. The median interval from initial diagnosis to surgical intervention was 2 (0–20) months.

## Discussion

In Japan, AAA screening has not yet been implemented, and its necessity has not been emphasized. In 2018, the Basic Act on Measures against Stroke and Cardiovascular Diseases was enacted to promote a 5-year plan for addressing stroke and cardiovascular diseases, including aortic diseases.^7)^ National data on the occurrence of AAA is not available, but non-interventional deaths, including out-of-hospital mortality due to ruptured AAA, are estimated to be as high as 3000 per year based on the Demographic Surveys conducted by MHLW and the JSVS annual report. This number is as large as one-sixth of the annual number of scheduled AAA surgeries.

The Society for Vascular Surgery (SVS) guideline described that “should AAA rupture occur, more than half of patients die before hospitalization or without treatment.”^8)^ In Japan, around a 70% mortality rate after the onset of AAA rupture is expected, as described in the introduction of this article, 10% of AAA operated on are for rupture, and their surgical mortality rate is around 15%–20%, which is extremely higher compared to 1% for scheduled AAA surgery. Diagnosis of AAA before rupture is essential, and public awareness of AAA is also essential for early detection. However, the present study confirmed that 80% of AAA were detected incidentally during examinations for other diseases, and health screenings (18.8%) in several pathways did not play a major role in the diagnosis of AAA.

The American Board of Expertise in Preventive Medicine and SVS guidelines recommend screening with AUS for men aged 65–75 years with a history of smoking as surveillance, with a 3-year follow-up for AAA of 3.0–3.9 cm and a 1-year follow-up for AAA of 4.0–4.9 cm, which should be followed up after 1 year.^8,9)^ In addition, European Society for Vascular Surgery (ESVS) 2024 Clinical Practice Guidelines stated that screening using AUS for the early detection of AAA is recommended in high-risk populations, specifically older than 65-year-old males who are former or current smokers and have other peripheral aneurysms (located at the iliac, femoral, and popliteal sites).^5,10–12)^ The present study similarly revealed that in patients with asymptomatic AAA, more common characteristics are elderly age, male gender, hypertension, and smoking. A family history of AAA is not so common, at 4.6%, and coronary artery disease was complicated in 22.0%, which is similar to previous reports.^13,14)^ Family history might not be a cue to AAA screening, but inspection of AAA with echocardiography for patients with coronary artery disease might be beneficial. It is also noteworthy that CKD was observed with a high prevalence of 30.3%, which is a common consideration in the management of AAA. The prevalence of AAA in CKD patients would suggest the necessity of AAA screening.

The Organisation for Economic Co-operation and Development (OECD) health care activities indicated that the number of CT scanners per 100000 population is around 100, which is the largest among those of the other OECD countries.^15)^ In terms of methods for AAA detection in the present study, although around 30%–40% of the AAAs were found during disease testing in the abdominal region, most of the AAAs were still found on CT scan (81.2%), and only 15.2% of AAAs were found on AUS. The detection accuracy of AAA by AUS is reported to be high, with a sensitivity of over 94% and specificity of over 98%, and is comparable to that of CT.^16)^ CT appears to be superior to AUS in terms of sensitivity, and CT can detect aneurysmal lesions not detected by AUS, but AUS has the advantage in terms of radiation exposure and cost.^17)^ Under the current insurance system in Japan, AUS costs 5300 yen and CT costs 9000 yen, which means that AUS costs about 60% less. In addition, since about 10% of asymptomatic AAAs were mainly treated as IAAs, close examination of the iliac region by AUS should be emphasized.

Previous randomized controlled trials (RCTs) revealed that the contribution of AAA screening to mortality reduction in men aged from 64 to 73 years old was maintained over time and screening was cost-effective.^18)^ The ESVS 2024 Clinical Practice Guidelines on the Management of Abdominal Aorto-Iliac Artery Aneurysms listed 4 further RCTs of population-based screening for AAA in men in the United Kingdom, Denmark, and Australia.^9)^ The odds ratios of AAA-related mortality of screened cohorts compared with non-screened cohorts were reported to be from 0.31 to 0.91 in each RCT, and the effect of AAA screening is clear. Similarly, SVS practice guidelines on the care of patients with AAA stated that the United States Preventive Services Task Force recommended one-time AUS screening for men aged from 65 to 75 years old with smoking history as a grade B recommendation and selective screening of 65- to 75-year-old men without smoking as grade C recommendation.^8,16)^ Therefore, the evidence for the cost-effectiveness of AAA screening in other countries is already sufficient.

Scheduled AAA surgery is extremely curable and would facilitate postoperative reintegration into society. Consequently, AAA screening using AUS for patients with risk factors of AAA (including elderly, male, hypertension, and smoking history) is in the public interest and is worth considering for implementation.

### Study limitations

Our study has some limitations, as follows: First, this multicenter, retrospective study on a specific cohort of patients was limited by the insufficient data on the devices used and differing institutional methods of measurement for aneurysmal diameter. Second, the details of the causes of mortality were not included in the data. Third, the details of surgical outcomes were not included in the data. Last, it is also unclear if patient factors, rather than aneurysm diameter, were the reason that surgical intervention was not indicated in some cases.

## Conclusion

Asymptomatic AAAs were more common among the elderly, men, people with hypertension, and smokers, making them suitable screening targets. Presently, most AAAs are detected incidentally during examinations for other diseases, and there is an urgent need to promote health screening. Most AAAs are diagnosed by CT, but AUS would be the most appropriate modality of AAA screening because of its accuracy, noninvasiveness, and low cost.

## Declarations

### Acknowledgments

We appreciate the support for the statistical settings and analyses from Dr Katsuhiro Omae, PhD, National Cerebral and Cardiovascular Center.

### Disclosure statement

All authors of this article have no conflicts of interest to disclose, and this research did not receive any specific grant from funding agencies in the public, commercial, or not-for-profit sectors.

### Author contributions

Study conception: all authors

Data collection: all authors

Analysis: YS, HM

Investigation: all authors

Manuscript preparation: YS, HM

Critical review and revision: all authors

Final approval of the article: all authors

Accountability for all aspects of the work: all authors.
